# Roles of *qseC* mutation in bacterial resistance against anti-lipopolysaccharide factor isoform 3 (ALF*Pm*3)

**DOI:** 10.1371/journal.pone.0286764

**Published:** 2023-06-02

**Authors:** Iyacoob Khunsri, Pinidphon Prombutara, Htut Htut Htoo, Supitcha Wanvimonsuk, Thanadon Samernate, Chindanai Pornsing, Sirinit Tharntada, Phattarunda Jaree, Vorrapon Chaikeeratisak, Kunlaya Somboonwiwat, Poochit Nonejuie

**Affiliations:** 1 Institute of Molecular Biosciences, Mahidol University, Nakhon Pathom, Thailand; 2 Center for Vaccine Development, Institute of Molecular Biosciences, Mahidol University, Nakhon Pathom, Thailand; 3 Omics Science and Bioinformatics Center, Faculty of Science, Chulalongkorn University, Bangkok, Thailand; 4 Center of Excellence for Molecular Biology and Genomics of Shrimp, Department of Biochemistry, Faculty of Science, Chulalongkorn University, Bangkok, Thailand; 5 Department of Veterinary Technology, Faculty of Veterinary Technology, Kasetsart University, Bangkok, Thailand; National Cheng Kung University, TAIWAN

## Abstract

Propelled by global climate changes, the shrimp industry has been facing tremendous losses in production due to various disease outbreaks, particularly early mortality syndrome (EMS), a disease caused by *Vibrio parahaemolyticus* AHPND. Not only is the use of antibiotics as EMS control agents not yet been proven successful, but the overuse and misuse of antibiotics could also worsen one of the most challenging global health issues—antimicrobial resistance. To circumvent antibiotic usage, anti-lipopolysaccharide factor isoform 3 (ALF*Pm*3), an antimicrobial peptide (AMP) derived from the shrimp innate immune system, was proposed as an antibiotic alternative for EMS control. However, prolonged use of AMPs could also lead to bacterial cross resistance with life-saving antibiotics used in human diseases. Here, we showed that ALF*Pm*3-resistant strains of *E*. *coli* could be induced *in vitro*. Genome analysis of the resistant mutants revealed multiple mutations, with the most interesting being a *qseC(L299R)*. A study of antibiotic susceptibility profile showed that the resistant strains harboring the *qseC(L299R)* not only exhibited higher degree of resistance towards polymyxin antibiotics, but also produced higher biofilm under ALF*Pm*3 stress. Lastly, a single cell death analysis revealed that, at early-log phase when biofilm is scarce, the resistant strains were less affected by ALF*Pm*3 treatment, suggesting additional mechanisms by which *qseC* orchestrates to protect the bacteria from ALF*Pm*3. Altogether, this study uncovers involvement of *qseC* mutation in mechanism of resistance of the bacteria against ALF*Pm*3 paving a way for future studies on sustainable use of ALF*Pm*3 as an EMS control agent.

## Introduction

In recent years, the situation of the world food security and starvation are rapidly worsening in every region across the globe [1–3]. Many factors such as social inequality, political conflicts, and climate changes, are accountable for the sharp drop in food production yield [3–5]. Particularly, negative impacts caused by climate abnormality were seen in all sectors of agricultural value chains from feed, farm and food [3]. Not only does the change in climate directly affect the production yield, as seen in the effects on certain crops around the world [5, 6], but unpredictable weather patterns also increase the likelihood of major disease outbreaks that severely decrease the production output of many agricultural products including farm animals [7].

Thailand is a littoral country situated between the Andaman Sea and the Gulf of Thailand with total coastal length of about 2,815 km. Thus, a vast area along the coastline is suitable for aquaculture—an essential player in Thai economy. Among all others, shrimp was listed as the top fishery product exported in terms of value, in the early 2020s [8]. However, in the past years, the shrimp industry has been facing increasing problems of disease outbreaks such as white spot syndrome virus (WSSV) disease [9, 10], yellow head virus (YHV) disease [11], and acute hepatopancreatic necrosis disease (AHPND) [12], causing a sharp drop in shrimp production. One of the forerunners, AHPND, also known as early mortality syndrome (EMS), a disease caused by a halophilic Gram-negative bacterium—*Vibrio parahaemolyticus* AHPND (*VP*-AHPND)—causes long-lasting and devasting wounds to the shrimp industry [12–14]. Since shrimp farming has become vital for Thai economy, protecting the shrimp from the perils of AHPND at all costs is essential. Commonly, shrimp farmers would use chemicals and antibiotics to control AHPND but, to date, there has been no confirmation that antibiotics can sustainably eliminate the disease [15]. Worryingly though, long-term use of antibiotics may induce antibiotic resistance in bacteria not only in the targeted pathogens but also in other bacteria in the environment [16, 17]. Furthermore, antibiotic resistant genes could also be transferred to other bacterial species including human pathogens, thereby uncontrollably spreading to the environment and ultimately leading to severe adverse effects on human health [17, 18].

In 2021, the World Health Organization declared antimicrobial resistance (AMR) as among the top 10 public health issues [19]. Since AMR is ubiquitous along the animal-human-environment interface [20, 21], unsurprisingly, the overuse of antibiotics in animals and livestock, as preventive measures of illnesses, and the misuse as growth promoters, has been identified as the major cause of AMR [15, 17, 22]. In order to find alternative ways to reduce the use of antibiotics, some have focused on exploiting the innate immune system of the organisms [23]. In particular, previous studies have identified antimicrobial peptides (AMPs) in the immune system of the shrimp that can inhibit the growth of *VP*-AHPND. ALF*Pm*3—the anti-lipopolysaccharide factor isoform 3, isolated from black tiger shrimp *Penaeus monodon*—is a member of the anti-liposaccharide factors (ALFs) family with a broad range of antimicrobial activities against both Gram-positive and Gram-negative bacteria, including *VP*-AHPND and *Vibrio harveyi*, a species that causes vibriosis [24–26]. In terms of mechanism of action, ALF*Pm*3 binds to the lipopolysaccharide (LPS) of the bacterial cell wall causing cell envelop damage followed by cell death [25, 26]. With its broad antibacterial activity, ALF*Pm*3 was proposed as a good candidate for replacing the use of antibiotics in shrimp farms [27]. However, the long-term use of ALF*Pm*3 against bacteria also raises concerns about the development of bacterial resistance. Studies on other AMPs have revealed that the use of cationic AMPs could induce resistance in a wide range of bacterial species including human pathogens [23, 28]. Therefore, it is reasonable to speculate that the ALF*Pm*3-resistant mechanisms might overlap with those of other antibiotics, leading to resistance development towards life-saving antibiotics.

Faced with the eminent risk of cross resistance between ALF*Pm*3 and antibiotics used for bacterial infection treatment in humans, we were compelled to investigate the possible mechanisms underlying ALF*Pm*3 resistance. First, we isolated three ALF*Pm*3-resistant strains of *E*. *coli* that are eight times more resistant to ALF*Pm*3 when compared to their sensitive counterpart. Genome analysis of these resistant mutants revealed that mutations were present in multiple genes, with the most interesting being that in the *qseC* gene, a gene involved in antibiotic stress response, among others. Next, the study of antibiotic susceptibility profile showed that the resistant strain harboring the mutation in the *qseC* gene showed higher degree of resistance towards cationic lipopeptide antibiotics; polymyxins. Accordingly, biofilm production assay revealed that the *qseC* mutation is also responsible for higher biofilm production in the presence of ALF*Pm*3. Lastly, single cell death analysis by microscopy revealed that the resistant strains at early-log phase were also less affected by the ALF*Pm*3 treatment, hinting on additional mechanisms by which QseC orchestrates to protect the bacteria from ALF*Pm*3.

## Results

### ALF*Pm*3-resistant *E*. *coli* isolation and their susceptibility against ALF*Pm*3

In order to examine if a long-term use of ALF*Pm*3 as an antibacterial agent could induce resistance, we first attempted to isolate ALF*Pm*3-resistant *E*. *coli*. Since the antibacterial activity of ALF*Pm*3 against the wildtype *E*. *coli* MC4100 used in this study (Table 1) had not been previously tested, we first determined the minimum inhibitory concentration (MIC) of ALF*Pm*3 against the bacteria and found that ALF*Pm*3 could inhibit the growth of *E*. *coli* MC4100 at 1 μM (Table 2). Then, we isolated ALF*Pm*3-resistant strains of *E*. *coli* by passaging the *E*. *coli* MC4100 in culture media supplemented with gradually-increasing concentrations of ALF*Pm*3, and successfully obtained three strains of ALF*Pm*3-resistant *E*. *coli*; PN1001, PN1002 and PN1004, the identities of which were confirmed by 16S rRNA analysis. Susceptibility test showed that the MIC of these strains are 8 times higher than that of the wildtype (Table 2).

**Table 1:** Table showing bacterial strains and plasmids used in this study.

	Characteristics	Reference
Bacterial strains		
*E*. *coli* MC4100	F^-^ *araD139 DlacU169 relA1 rpsL150 thi mot flb5301 deoC7 ptsF25 rbsR*	Laboratory collection
*E*. *coli* JP313	MC4100 *araΔ*714	Pogliano *et al* (1997) [65]
IC1001	*E*. *coli* JP313 *qseC*::*camR*	This study
Plasmids		
pTargetF	*pMB1 aadA* sgRNA-*pMB1*, s*pecR*	Addgene: 62226
pCas	*repA101* (Ts) *kan P*_*cas*_*-cas9 P*_*araB*_*-Red lacI*^*q*^ *P*_*trc*_*-*sgRNA-*pMB1*	Addgene: 62225
pTargetF-*qseC*	*pMB1 aadA* sgRNA-*qseC*, s*pecR*	This study

**Table 2:** Table showing the MICs of ALF*Pm3* in *E*. *coli* strains and their mutated genes.

Strains	MIC (μM) of ALF*Pm*3	Mutant alleles		
		Gene	Nucleotide	Amino acid
MC4100	1	-	-	-
PN1001	8	Intergenic	A2323955T	-
		Hypothetical protein	c.C3995906T	p.Cys143Tyr
		*ddlB*	c.C103024T	p.Asp264Asp
PN1002	8	Intergenic	C921168A	-
		*cpdB*	c.T4320752C	p.Val553Val
PN1004	8	*qseC*	c.A3611241C	p.Leu299Arg
IC1001	4	*qseC*::*camR*	-	-

### Genome analysis of ALF*Pm*3-resistant *E*. *coli*

In order to examine the genes responsible for ALF*Pm*3 resistance, once the resistant strains were obtained, whole genome sequencing of all three strains were carried out in comparison with the MC4100 strain. In total, six mutations were detected—two in the intergenic region and four in the coding region (Table 2). In particular, PN1001 harbors one mutation in an intergenic region (A2323955T) and two mutations in a coding region including c.C3995906T encoding for hypothetical protein and c.C103024T that causes silent mutation in *ddlB* gene. One intergenic mutation (C921168A) and, one silent mutation c.T4320752C in *cpdB* gene, were found in PN1002. The genome of PN1004 contains only one mutation at c.A3611241C which the gene is annotated as *qseC* gene.

Among the mutations in the coding regions, *ddlB* and *qseC* genes are of interest due to their involvement in bacterial resistance against other antibiotics from previous studies. For example, the mutation in *ddlB*, a gene encoding for D-alanine-D-alanine ligase (VanA) that is involved in the peptidoglycan biosynthesis of bacteria, was reported to be involved in vancomycin resistance, by altering the substrate of the VanA, thereby preventing vancomycin from attaching to the substrate on the bacterial cell wall [29–31]. However, the fact that the mutation in *ddlB* found in this study is a silent mutation makes it less desirable for further study. In contrast, a missense mutation found in the *qseC* gene (L299R) of PN1004 is particularly interesting. QseC is part of the two-component regulatory system quorum sensing in bacteria, including *E*. *coli* [32]. The quorum sensing plays an important role in cell-cell signaling in bacteria that regulates *pmrB* gene that has been reported to be involved in bacterial resistance against the cationic antimicrobial peptide antibiotic, polymyxin [28, 33]. Altogether, the whole genome analysis of all resistant mutants revealed multiple mutated genes, some of which were previously shown to affect susceptibility of the bacteria to antibiotics.

### ALF*Pm*3-resistant strains harboring *qseC* mutations showed higher resistance to lipopeptide antibiotics, polymyxin and colistin

The presence of an antibiotic resistance-related mutation in the resistant strains urged us to further investigate if the antibiotic susceptibility profiles of these mutant strains were altered. We examined the MIC of all mutants against 11 different antibiotics from five major classes of antibiotics including DNA replication inhibitors, RNA transcription inhibitors, protein translation inhibitors, cell wall synthesis inhibitors, and membrane disrupting agents. The result showed that the strains PN1001 and PN1002 did not exhibit drastic changes in their antibiotic susceptibility profile when compared to that of wildtype MC4100 (Table 3). Only slight, two-fold changes were seen in some antibiotics such as kanamycin and nalidixic acid, suggesting that mutations found in PN1001 and PN1002 do not contribute to the significant changes in their susceptibility profiles against all 11 antibiotics tested in this study.

**Table 3:** Table showing the antibiotic susceptibility profile of *E*. *coli* strains used in this study.

Antibiotics	MIC (μg/ml) in *E*. *coli* strains				
	MC4100	PN1001	PN1002	PN1004	IC1001
Protein translation inhibitors					
Gentamicin	2	2	1	2	-
Kanamycin	4	8	8	8	-
Tobramycin	2	4	1	4	-
Chloramphenicol	4	4	4	4	-
DNA replication inhibitors					
Ciprofloxacin	0.008	0.008	0.008	0.002	-
Nalidixic acid	8	4	4	4	-
RNA transcription inhibitor					
Rifampicin	8	8	8	8	-
Cell wall synthesis inhibitors					
Amoxicillin	4	4	4	4	-
Meropenem	1	0.5	1	0.5	-
Membrane disrupting agents					
Polymyxin B	0.25	0.25	0.25	8	-
Colistin	0.5	0.25	0.25	16	1

**Fig 1:**
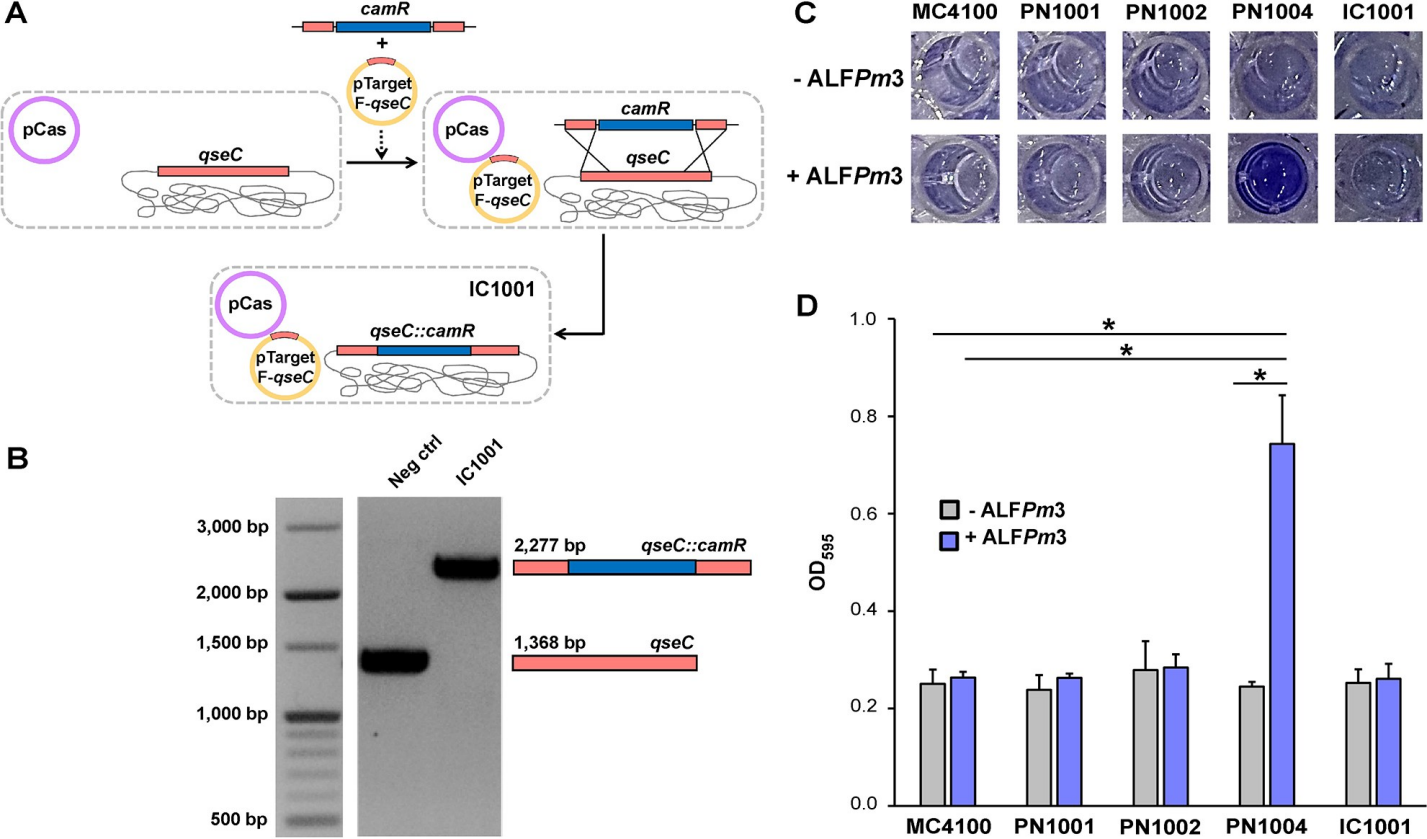
*E*. *coli* harboring *qseC(L299R)* mutation produces higher biofilm under ALF*Pm*3 stress. (A) Construction of IC1001 strain via CRISPR-Cas two-plasmid system. (B) Confirmation of the presence of *qseC*::*camR* in IC1001 strain. Neg ctrl is a representative of unsuccessful CRISPR-engineered clone containing wild type *qseC* gene. (C) A representative result of biofilm formation of tested bacterial strains with or without ALF*Pm*3 (half MIC). (D) Quantification of biofilm formation after 24 hours incubation with or without ALF*Pm*3 supplement. The error bars illustrate the standard deviations of three separate experiments. The statistical significance using two-tail student t-test (*P < 0.05).

Although susceptibility profile of PN1004 harboring the *qseC(L299R)* mutation did not drastically alter DNA replication, RNA transcription, protein translation, and cell wall synthesis inhibitors tested, it showed 32 times higher resistance to a group of membrane disrupting agents; polymyxin B and colistin, when compared to that of MC4100. This result highlights the importance of *qseC* mutation in bacterial resistance against both ALF*Pm*3 and cationic peptide polymyxin antibiotics. Spurred by this result, we further investigated if the *qseC* gene was indeed responsible for the resistance to ALF*Pm*3 and colistin. Through genome engineering, the *qseC* gene in *E*. *coli* MC4100 was replaced by chloramphenicol resistant gene *camR*, to generate a new strain of *E*. *coli* harboring *qseC*::*camR* designated as IC1001 (Fig 1A and 1B). Notably, the MICs of ALF*Pm*3 and colistin in IC1001 are also higher when compared to MC4100 (Tables 2 and 3), indicating that *qseC* deletion is also responsible for higher degree of bacterial resistance against ALF*Pm*3 and colistin. However, its resistant level is yet lower than that of the original PN1004 strain: 2- and 16-fold lower in ALF*Pm*3 and colistin, respectively (Tables 2 and 3), suggesting that *qseC*::*camR* is not equivalent to *qseC(L299R)* in terms of promoting the resistant level of the bacteria against ALF*Pm*3 and colistin.

### *qseC* mutation resulted in higher biofilm production of the bacteria under ALF*Pm*3 stress

Next, we examined what possible phenotypic responses the bacteria harboring *qseC* mutations might employ to protect themselves against ALF*Pm*3. Previous studies have shed light onto the relationship between two-component signaling system QseBC and PmrAB, showing the involvement of the system in biofilm formation [32, 34]. We, thus, tested if the *qseC* mutations are affecting biofilm formation, and eventually leading to antibiotic resistance. The result showed that, in an absence of ALF*Pm*3, no significant difference in biofilm production was detected in all strains tested. However, when supplemented with sub-MIC level of ALF*Pm*3, the amount of biofilm generated from PN1004 was significantly higher than that of MC4100 (Fig 1C and 1D). Notably, the deletion of *qseC* gene (*qseC*::*camR)* did not significantly alter biofilm production of the bacteria (IC1001) either with or without ALF*Pm*3 supplement, suggesting that *qseC(L299R)*, and not the *qseC*::*camR*, is responsible for the higher production of the biofilm of the bacteria under ALF*Pm*3 stress.

### Single cell death analysis by microscopy showed that *qseC(L299R)* and *qseC*::*camR* strains are less sensitive to ALF*Pm*3 at early timepoint

**Fig 2:**
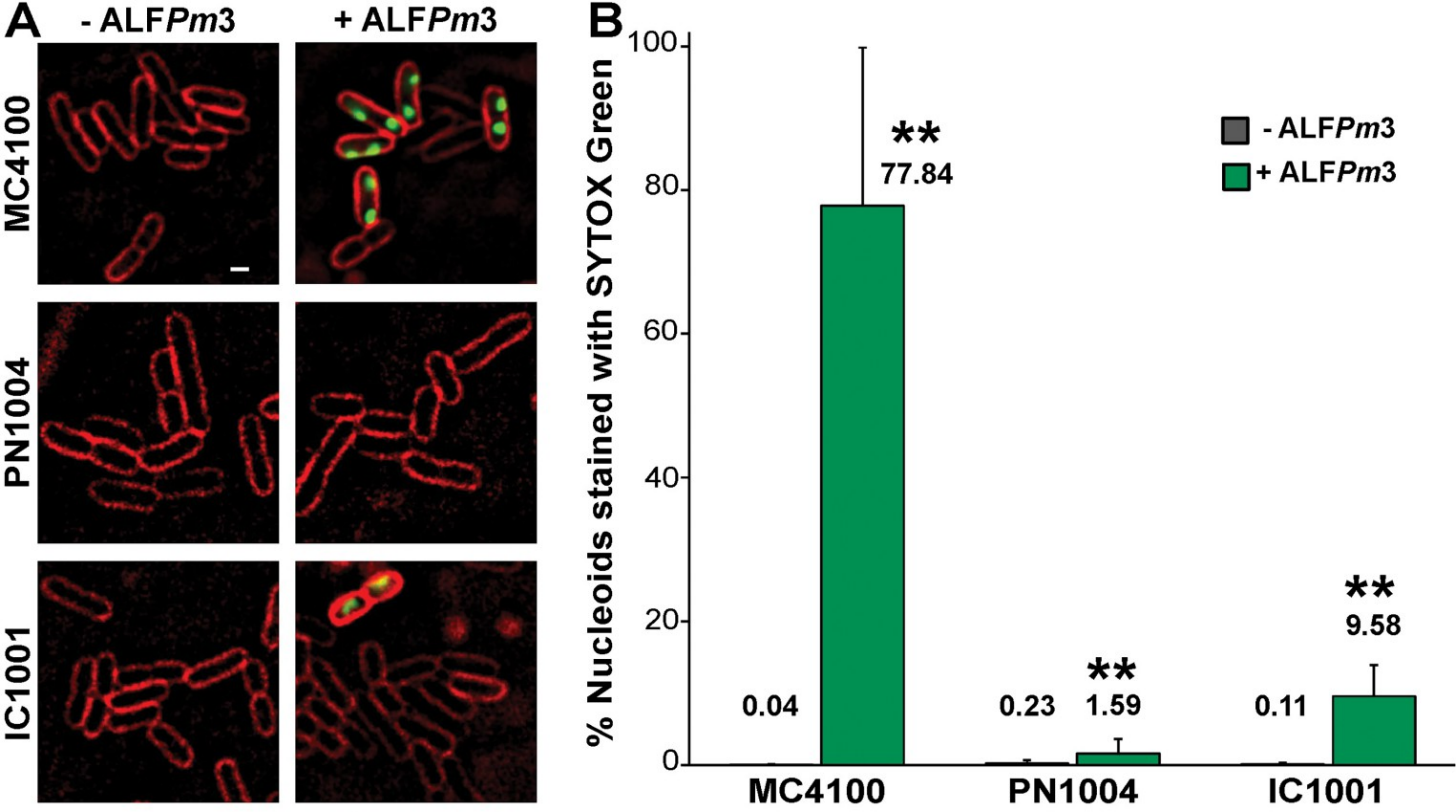
Single cell death analysis reveals protective effect of *qseC* mutations against ALF*Pm*3 at early timepoint. (A) Representative images of MC4100, PN1004 and IC1001 strains of *E*. *coli* that were incubated with and without 1 μM ALF*Pm*3 for 1 hour then stained with 1 μg/ml FM4-64 (red) and 0.5 μM SYTOX Green (green) fluorescent dyes. Scale bar represents 1 μm. (B) A graph showing the difference in the % of nucleoids that are stained with SYTOX Green between treatment conditions with and without ALF*Pm*3, in MC4100, PN1004 and IC1001 strains. The % of nucleoids stained are indicated. The error bars indicate standard deviations from 3 independent experiments as detailed in the materials and methods. ** *P* < 0.001.

It is well known that, under the limited nutrient environment or stress, biofilm production of the bacteria occurs as a means of survival response [35–37]. The fact that IC1001, which is four times more resistant to ALF*Pm*3 than MC4100, showed no significant increase in biofilm production urged us to investigate whether or not, at the early stage of bacterial growth where biofilm production is limited, bacteria with *qseC* mutations are protected from ALF*Pm*3. Thus, we performed fluorescence microscopy to investigate the levels of single cell death in MC4100, PN1004 and IC1001 strains upon 1 μM ALF*Pm*3 treatment. When treated with ALF*Pm*3, 77.84% of wildtype MC4100 cells were stained with SYTOX Green, a fluorescent dye that indicates cell death since it is unable to enter cells with intact membranes [38], whereas only a 1.59% and 9.58% of PN1004 and IC1001 cells, respectively, were stained (Fig 2). This suggests that both resistant strains were protected from ALF*Pm*3 at the early stage of the growth when biofilm is still absent. Notably, in accordance with the difference in MIC levels of the resistant strains, PN1004 which possess higher MIC of ALF*Pm*3, was significantly less affected by ALF*Pm*3 treatment in the single cell death analysis when compared to IC1001.

## Discussion

In an era of antibiotic resistance, novel antibiotics are urgently needed in order to combat antibiotic resistant pathogens. However, searching for new antibiotics is notoriously difficult [39, 40]. Thus, in recent years, antibiotic alternatives, whose potential have not yet been fully exploited, have been in the spotlight, due to their diversity and abundance [41]. One of the most promising antibiotic alternatives are antimicrobial peptides (AMPs) since they were previously prioritized in Tier 2 approaches, where breakthrough insights in systemic therapy are emerging [40, 42]. Albeit their vast diversity and broad antibacterial activity, using AMPs as therapeutic agents also raised concerns about the impact on the innate immune system of higher eukaryotic organisms, due to the similarity between the therapeutic AMPs and those of the eukaryotes [43]. Also, many studies worryingly revealed that resistance against AMPs shared some similarity with the resistance to certain antibiotics [43]. An interesting example would be that in clinical isolates of *Acinetobacter baumannii* that were exposed to colistin, cross-resistance to AMPs, including a human-derived peptide LL-37, was observed [44]. Thus, understanding the mechanism of resistance of the bacteria against AMPs is vital for the sustainable use of AMPs as antibiotic alternatives; thereby truly alleviating the worsening situation of AMR.

Here we demonstrated that ALF*Pm*3-resistant *E*. *coli*, isolated in this study, harbors various mutations in the coding regions that might be associated with antibiotic resistance. One of the limitations worth mentioning is that this study focused only on the role of *qseC* gene mutation found in PN1004. Thus, the characteristics of ALF*Pm*3 resistance of PN1001 and PN1002 were not fully investigated. Particularly, the possible role of missense mutation at c.C3995906T which was annotated as a hypothetical protein in ALF*Pm*3 resistance was not included in this study. Many studies have identified myriad of mutated hypothetical proteins that are involved in antibiotic resistant mechanism of bacteria [45, 46]. Thus, it is interesting to further explore the role of the hypothetical protein in ALF*Pm*3 resistance that might lead to a better understanding of how bacteria become resistant to ALF*Pm*3.

Apart from mutations in the coding regions, mutations in the intergenic regions of both PN1001 (A2323955T) and PN1002 (C921168A) found in this study are also intriguing. It is possible that mutations in intergenic areas may affect neighboring genes by altering regulatory components that govern the target gene, despite the intergenic region’s distance from the target gene. A previous study found that, A2323955T mutation, located near the *fabB* gene, which encodes for 3-oxoacyl-ACP synthase in fatty acid synthesis in lipid metabolism, contributed to the overexpression of the *fabB* gene resulting in the upregulation of the efflux pump and hence, bacterial resistance against tialactomycin [47]. In this study, intergenic region mutation at C921168A in PN1002 is also a promising candidate for further studies since it is located near *ompA* gene that encodes for outer membrane protein A. OmpA is a well-known protein that is involved in various antibiotic resistance pathways of the bacteria. For example, a previous study showed that *E*. *coli* resistant to tetracycline displayed an increase in the expression of *ompA* [48]. Thus, whether or not these intergenic mutations are responsible for ALF*Pm*3 resistance needs further investigations.

Biofilm production has been linked to bacterial resistance against various antibiotics in many studies [49, 50]. It has been shown that biofilm formation also contributed to AMP resistance by electrostatic repulsion from biofilm capsule polymers [51], and sequestration of negatively charged AMPs by the positively charged biofilm polysaccharides and proteolytic breakdown of AMPs [52]. Thus, it is not unexpected that the ALF*Pm*3-resistant bacteria with higher biofilm production confer resistance to other peptide-based antibiotics that share similar MOA such as the lipopeptide polymyxin. Regarding *qseC* mutation, we showed that not only is the bacteria harboring *qseC(L299R)* mutation more resistant to ALF*Pm*3 and colistin, but it also produces higher amount of biofilm in the presence of ALF*Pm*3. In support of these findings, many previous studies have demonstrated that the *qseC* gene, which belongs to the PhoPQ family, was involved in virulence and biofilm formation of bacteria [49, 53]. It is interesting to note that even though the strain harboring *qseC*::*camR* mutation is also resistant to ALF*Pm*3, it did not show significant changes in biofilm production, either in the absence or the presence of ALF*Pm*3. The finding is slightly in contrast with a previous study where deletion of *qseC* gene resulted in impaired biofilm formation, among other stresses, in *Haemophilus parasuis* [49].

It is also worth noting that, even though we showed that *qseC(L299R)* mutation in PN1004 resulted in higher biofilm production upon ALF*Pm*3 treatment at 24 hours, there is no direct evidence showing that biofilm formation is the sole protective mechanism of the resistant bacteria from ALF*Pm*3. It is possible that the higher production of biofilm at 24 hours, contributed to higher MIC data which was also collected at 24 hours timepoint. However, the higher biofilm formation at this time point cannot fully explain the resistance characteristics of the bacteria at the early stage of growth as seen in our single cell death analysis. At this early stage of cell growth (early-log phase), where biofilm production is limited, we found that the bacteria harboring *qseC(L299R)* or *qseC*::*camR* mutations were also protected from ALF*Pm*3, which resulted in significantly lower number of dead cells. This finding suggests that *qseC* might exert its resistant mechanism toward ALF*Pm*3 via different cellular pathways, that have yet to be determined in future studies. It is possible that *qseC* mutation might alter the membrane envelop properties of the bacteria making the resistant bacteria less susceptible to the cationic peptide as seen in other resistance mechanisms against various peptide-based antibiotics [54, 55]. Further studies regarding mechanism of resistance via *qseC* could provide an insight into how the gene might be involved in antimicrobial peptide resistance of the bacteria.

## Materials and methods

### Bacterial strains and plasmids

Complete details on the bacterial strains and plasmids used in this study, along with its characteristics and sources are listed in Table 1.

### Production and purification of the recombinant ALF*Pm*3 protein

The recombinant ALF*Pm*3 (rALF*Pm*3) protein was produced in the yeast *Pichia pastoris* according to a previous study [56]. Briefly, a single colony of *P*. *pastoris* containing ALF*Pm*3 was grown overnight in YPD broth medium at 30°C. Later, the overnight culture was inoculated into BMGY medium and grown at 30°C until an OD_600_ of 4–6. The cell was induced for protein production by being transferred to culture in BMMY medium. Furthermore, methanol (100%) was added every 24 hours to a final concentration of 0.5% (v/v). At day 2 post methanol induction, the supernatant, which is crude rALF*Pm*3, was collected. To purify rALF*Pm*3 protein, the crude supernatant was diluted 1:1 with the starter buffer (20 mM Tris–HCl, 200 mM NaCl, pH 7.0), and then purified by a strong cation exchange chromatography, SP Sepharose High-Performance column, (GE Healthcare). Elution was then performed with the elution buffer (20 mM Tris–HCl, 1 M NaCl, pH 7.0) and dialyzed overnight against sterile deionized water at 4°C to reduce the salt. The purified rALF*Pm*3 was analyzed by SDS-PAGE using Coomassie blue staining and by western blot analysis using a specific antibody. The purified protein was kept at -20°C until used.

### MIC determination

MIC is the lowest concentration that can successfully inhibit the growth of the bacteria, and was determined by broth microdilution method [57]. Briefly, overnight cultures of single colonies of bacteria were grown in fresh Luria-Bertani (LB) broth on a roller at 50 rpm, at 30°C. Early log phase cultures were further diluted in LB broth and added to wells of a 96-well plate that contained 2-fold serial dilutions of ALF*Pm*3 or antibiotics in LB broth, to obtain 100,000 CFU/ml. The plate was then incubated at 30°C while shaking at 250 rpm for 24 hours. The MIC was interpreted by visual observation as the lowest concentration of the drug that can inhibit the growth of the bacteria.

### ALF*Pm*3-resistant bacteria selection and confirmation

ALF*Pm*3 resistance induction in *E*. *coli* was done by serial passage method [58]. Overnight cultures of single colonies of *E*. *coli* MC4100 were diluted in fresh LB broth and grown on a roller at 50 rpm, at 30°C. Early log phase cultures were further diluted in LB broth and cultured in a 96-well plate in the presence of sub-MIC levels of ALF*Pm*3 and incubated at 30°C for 24 hours. The cultures were then passaged in higher concentrations of ALF*Pm*3 and further incubated for 24 to 48 hours until the bacteria can successfully grow in this higher concentration of ALF*Pm*3.

All of resistant isolates were confirmed by 16S rRNA analysis. Briefly, *E*. *coli* were cultured in LB broth at 30°C overnight prior to cell collection and genomic DNA extraction using FavorPrep^TM^ Tissue Genomic DNA Extraction Mini Kit. The 16S rRNA was amplified with primers BSF8/20 (5’ AGAGTTTGATCCTGGCTCAG 3’) and REVB (5’ GGTTACCTTGTTACGACTT 3’) and purified with GenepHlow^TM^ Gel/PCR Kit. 16S rRNA’s DNA sequence were assessed on https://blast.ncbi.nlm.nih.gov/Blast.cgi.

### Whole genome sequencing and mutated gene analysis

Whole genome sequencing (WGS) and analysis of both wild type and resistant strains were carried out by the Omics Science & Bioinformatics Center at Chulalongkorn University. Briefly, the raw data sequence performance was assessed using FASTQC (v0.11.9), and irrelevant data was removed using Trim Galore (v0.6.2). The trimmed pieces were then assembled via SPAdes (v3.14.1). SNIPPY software (v4.3.6) was utilized to assess the mutations in resistant clones and to compare them with the reference *E*. *coli* K-12 sub-strain—MC4100 (NCBI txid:1403831). Prokka (v1.13.4) annotated the genomic DNA sequences with mutations in order to determine characteristics of the altered genes. Finally, putative mutant genes of resistant clones were identified by comparing with those of the wild type. All mutations shown in Table 1 were confirmed by Sanger sequencing.

### Bacterial genome engineering

To replace wildtype *qseC* with chloramphenicol resistant gene (*camR*), we performed CRISPR-based bacterial genome engineering using a two-plasmid system; pCas and pTargetF, according to previous studies [59, 60]. Briefly, gibson assembly was used to insert the N20 sequence, which includes the PAM sequence for the target locus of *qseC* gene, into pTargetF to generate pTargetF-*qseC*. Also, the homologous DNA template consisting of *camR* flanked by *qseC* homology was generated. *E*. *coli* JP313 harboring pCas was then transformed with 400 ng of homologous DNA template and 100 ng of pTargetF-*qseC by* electroporation. Transformants were selected on LB agar supplemented with 50 μg/ml each of spectinomycin and kanamycin, and 5 μg/ml chloramphenicol.

### Biofilm formation assay

The biofilm forming capability of bacteria was evaluated based on previous studies [61, 62]. Briefly, overnight cultures of bacteria were diluted 1:100 into fresh LB broth and grown until the OD_600_ reached 0.4. The cells were aspirated into a 96-well plate and then treated with or without half MIC of ALF*Pm*3 at 37°C for 24 hours. The biofilms were rinsed with water and PBS before being dyed with 150 ml of 0.1% crystal violet for 30 minutes. One hundred microliters of 30%(v/v) acetic acid were added to each well, which was subsequently transferred to be analyzed by a spectrophotometer at a wavelength of 595 nm (S1 Table).

### Fluorescence microscopy

Well separated single colonies of *E*. *coli* MC4100, PN1004 and IC1001 were inoculated in LB and grown overnight on a roller at 30°C. Overnight cultures were diluted in fresh media and grown until early log phase, until OD_600_ of 0.2 was reached. All strains were then treated for 1 hour with 1 μM of ALF*Pm*3 and accompanied by untreated controls. The cells were then stained with fluorescent dyes—1 μg/ml FM4-64, 2 μg/ml DAPI and 0.5 μM SYTOX Green. The cells were then harvested by centrifugation and concentrated by resuspending in LB at 1/10 of the original volume, after which 3 μl was loaded onto an agarose pad containing 1.2% agarose in 10% LB, the cover slip applied and then subjected to fluorescent microscopy on DeltaVision^TM^ microscope, with consistent parameters throughout all experiments. Excitation/emission wavelengths of the fluorescent dyes are 575/679 nm, 390/435 nm and 475/525 nm for FM 4–64, DAPI and SYTOX Green, respectively.

### Image analysis

Images obtained from the microscope were first preprocessed on Fiji software [63] and then subjected to analysis on CellProfiler software [64] version 4.3.1. The nucleoid outline, stained by the fluorescent dye DAPI, was used to define the area at which to measure the SYTOX Green intensity, after which the background intensity was subtracted to obtain the actual SYTOX intensity of individual cells. SYTOX Green intensity was measured in the unadjusted image obtained from fluorescence microscopy.

### Statistical analysis of single cell death calculations

Statistical analysis for single cell death calculations was performed on Microsoft Excel version 16.59. Statistical analysis was carried out on 3 independent experiments, each experiment comprising of; 5 images per strain, each having 40 to 300 nucleoids, for–ALF*Pm*3 conditions; and 7 to 11 images per strain, each with 30 to 400 nucleoid outlines, for + ALF*Pm*3 conditions. The percent of SYTOX Green is the percent of cells, per strain of bacteria, that are higher than 3 times the mean of SYTOX intensity of the corresponding untreated controls. Statistical significance was from two-tailed Student’s t-test, comparing between–ALF*Pm*3 and + ALF*Pm*3 conditions (** *P* < 0.001), error bars represent standard deviation (Fig 2, S2 Table).

## Supporting information

S1 Table Minimal dataset for biofilm assay showing absorbance at 595 nm for all strains with and without ALF*Pm*3 treatment (Minimal data for Fig 1D). (XLSX)

S2 Table The percent of nucleoids stained with SYTOX Green that are more than 3 times the mean SYTOX intensity of the untreated (- ALF*Pm*3) condition per strain of bacteria, together with P-values (Minimal dataset for Fig 2B). (XLSX)

S1 Raw image Raw image (agarose gel) for Fig 1B. (PDF)
