# Supplementary material for: Roles of qseC mutation in bacterial resistance against anti-lipopolysaccharide factor isoform 3 (ALFPm3)
Source: PLoS One. 2023 Jun 2;18(6):e0286764. doi: 10.1371/journal.pone.0286764 (PMC10237662; doi:10.1371/journal.pone.0286764)
Supplement: S1 Raw image — (PDF) [file pone.0286764.s003.pdf]

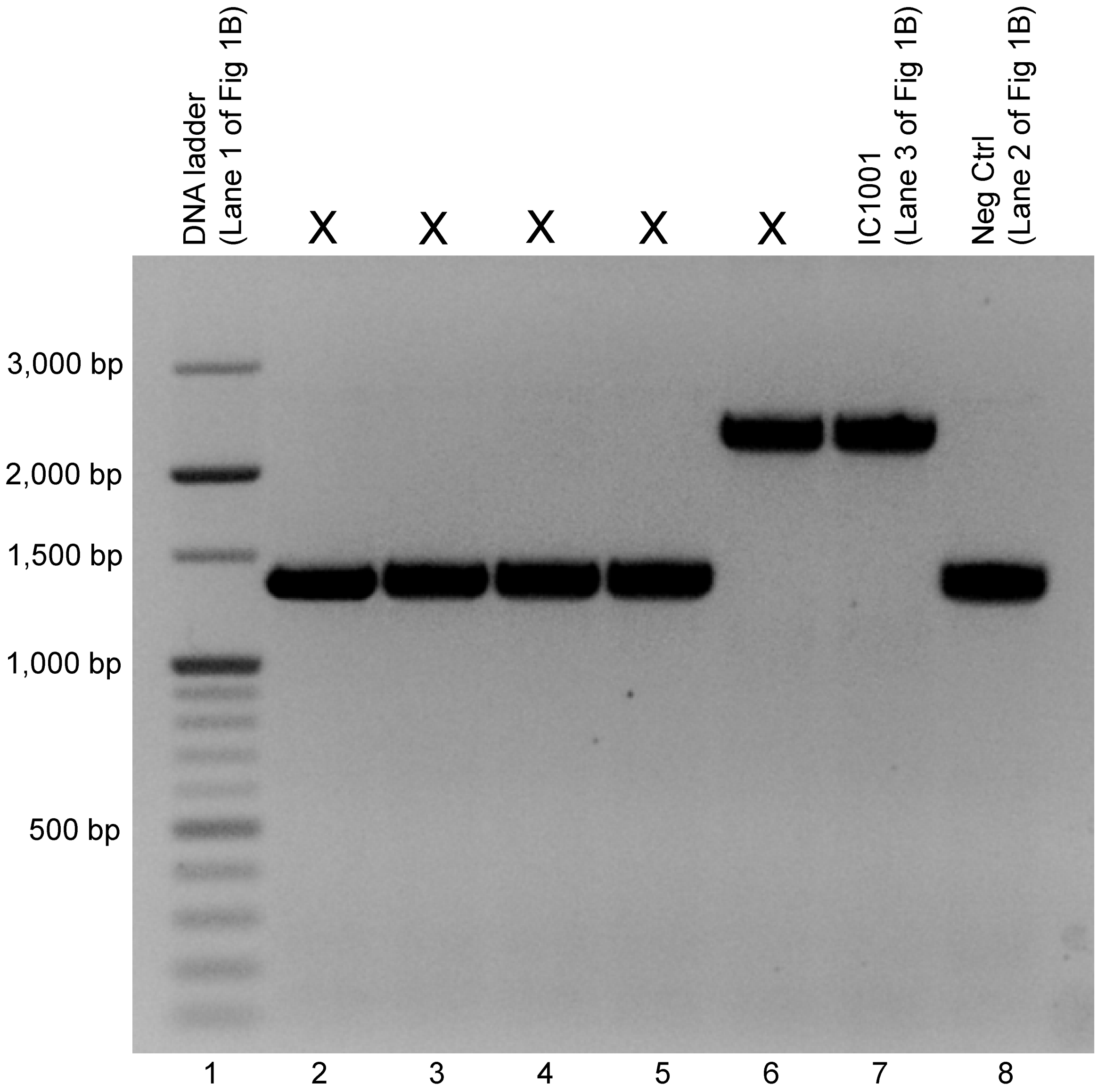

S1 Raw image. Raw image (agarose gel) for Fig 1B.

Raw image of Fig 1 panel B showing unmanipulated, uncropped image of agarose gel, stained with ethidium bromide, and documented on SYNGENE Gel Documentation system. X (lanes 2 – 6) indicates that these lanes are not included in the final figure (Fig 1B). Lane 1 is the DNA marker that is shown on Fig 1B. Lane 8 and lane 7 correspond to lane 2 and 3 of the final figure (Fig 1B).
